# Feasibility and stability of left bundle branch pacing in patients after prosthetic valve implantation

**DOI:** 10.1002/clc.23413

**Published:** 2020-07-01

**Authors:** Jincun Guo, Linlin Li, Guosheng Xiao, Xinyi Huang, Qiang Li, Yan Wang, Binni Cai

**Affiliations:** ^1^ Division of Cardiology Xiamen Cardiovascular Hospital, Xiamen University Xiamen Fujian China; ^2^ Division of Echocardiography Xiamen Cardiovascular Hospital, Xiamen University Xiamen Fujian China

**Keywords:** left bundle branch pacing, permanent pacemaker implantation, physiological pacing, prosthetic valves

## Abstract

**Background:**

Left bundle branch pacing (LBBP) has emerged as a promising pacing modality for preventing pacing induced cardiomyopathy in patients complicated with conduction abnormalities (CAs) after prosthetic valve (PV) implantation.

**Objective:**

The present study aimed to evaluate the safety and feasibility of LBBP in this patient population.

**Methods:**

LBBP was attempted in 20 patients complicated with atrioventricular block after PV implantation. Surface, intracardiac electrical measurements, and echocardiographic data were documented. Lead parameters and complications were routinely tracked at implantation and each follow‐up visit.

**Results:**

LBBP was successful in 90% (18/20) participants. The paced QRS duration and the stimulus to left ventricular activation time were 106.8 ± 6.8 ms and 65.5 ± 5.4 ms, respectively. Left bundle branch (LBB) potential was recorded in 61.1% (11/18) patients who succeeded in LBBP. During the procedure, the mean unipolar myocardium capture threshold was 0.51 ± 0.15 V@0.4 ms while the unipolar bundle capture threshold was 0.84 ± 0.51 V@0.4 ms. The mean fluoroscopic exposure time and the radiation dose were 13.0 ± 9.2 min and 81.7 ± 8.3 mGy, respectively. The average follow‐up period was 10.4 ± 5.9 months (range 3‐23 months). Pacing parameters remained stable and no significant lead‐related complications occurred during the whole observation period.

**Conclusions:**

LBBP was safe and feasible in patients with PVs. Acceptable and stable pacing parameters could be expected during the procedure and the follow‐ups.

## 1 INTRODUCTION

Atrioventricular heart block is a common complication in patients received prosthetic valve (PV) implantation. The incidences ranging from 5% to 30% in patients underwent transcatheter aortic valve replacement (TAVR), approximately 5% to 10% in patients received surgical aortic valve replacement (SAVR), about 27% in patients following surgical tricuspid valve replacement (TVR) and 4.5% to 10.5% in patients underwent mitral valve repairmen (MVr) or mitral valve replacement (MVR).^1^, ^2^, ^3^, ^4^ It is widely known that conduction abnormalities (CAs) and right ventricular pacing (RVP) subsequently to PV implantation may induce electrical asynchrony, cardiac contraction asynchrony, atrial fibrillation (AF), heart failure, and early or late all‐cause mortality during short‐term or long‐term observation.^5^, ^6^ Left bundle branch pacing (LBBP) is a promising physiological pacing modality which has been proved to maintain electrical and mechanical synchrony and translate better hemodynamic effect compared with traditional RVP during the acute period in previous studies.^7^, ^8^ The objective of this study was to investigate the safety and feasibility of LBBP in patients had PV implantation.

## 2 METHODS

### 2.1 Patient selection

This was a retrospective observational study fulfilled in Xiamen Cardiovascular Hospital, Xiamen University. Consecutive patients had PV implantations and subsequent atrioventricular heart block induced permanent pacemaker (PPM) implantations were recruited from February 2018 to December 2019. Patients with a prior history of PPM or bradycardia due to sick sinus disease were excluded. For retrospective analysis of clinically acquired data, the institutional review board waived the need of written patient informed consent. All data used for this study were acquired for clinical purposes and were handled anonymously.

### 2.2 Lead implantation

LBBP was performed using a transventricular septal method which has been described elsewhere.^9^ His region was mapped during the procedure and the locations of PVs served as a landmark for His region recognition. The lead (model 3830, 69 cm, Medtronic Inc., Minneapolis, Minnesota) with the sheath (C315 HIS, Medtronic Inc.) was advanced to the anterior‐inferior zone of His region, paced with high output (5.0 V@0.4 mv) and screwed into the septum perpendicularly once paced morphology of V1 presented a “W” pattern. The criteria for a successful LBBP procedure included: (a) paced QRS as a RBBB pattern; (b) with an LBB potential except for those with complete left bundle branch block (CLBBB); (c) stimulus to left ventricular activation time (stim‐LVAT) shortening abruptly with increasing output or remained shortest and constant both at low and high outputs; (d) evidence of selective LBBP; and (e) evidence of direct LBB capture (not routinely used in clinical practice).^10^ A LBB capture threshold of ≤2.0 V@0.4 ms and a myocardium capture threshold of ≤1.0 V@0.4 ms were considered acceptable in the current study. A dual‐lead method was applied in patients experienced difficulties during the LBBP procedure.^11^ In brief, the first fixed lead was kept as an anatomical landmark if the threshold was unacceptable or the stim‐LVAT was unacceptable, while a second 3830 lead was advanced to the adjacent area of the first lead to seek a site with better pacing parameters or stim‐LVAT. The LBBP lead was connected to the ventricular port while the atrial lead, which was usually fixed in the right atrial appendage, was connected to the atrial port. Additionally, a dual‐chamber pacemaker was implanted for patients with a sinus rhythm. For patients with AF, only the LBBP lead and single‐chamber pacemaker were implanted.

### 2.3 Surface, intracardiac electrocardiographic, and clinical data

Baseline patient characteristics were collected retrospectively. Twelve‐lead ECGs and echocardiographic data were obtained at least 12 to 24 hours prior to the procedure and at each follow‐up visit for each patient. Patients were followed up at predischarge, 1, 3, 6, and 12 months after the procedure. The intracardiac and surface electrograms were recorded by the GE CardioLab Electrophysiology recording system (GE Healthcare Inc., Marlborough, Massachusetts) at 100 mm/s and were used to obtain the baseline cardiac rhythm, various intervals, and CAs. The mean value obtained from at least three complexes was used for the subsequent analysis. The intracardiac electrogram (IEGM) was recorded at implantation and the paced QRS duration (pQRSD), stim‐LVAT and potential to ventricular interval (PVI) were recorded in sequence. The pQRSD was defined as the length of time from the onset of the first deviation from baseline for selective LBBP and from the onset of steepest deflection for nonselective LBBP to the end of the QRS complex in the 12 leads. The selective LBBP was defined as the direct activation of LBB with a discrete component between the stimulus and onset of QRS complex under threshold output while the nonselective LBBP captured adjacent local myocardial tissue resulting in a delta wave before the QRS complex. The stim‐LVAT was defined as the length of time from the pacing stimulus to the peak of R‐wave in lead V5 or V6. The PVI was measured from the LBB potential to the onset of QRS complex.

Lead parameters were documented at each follow‐up visit. Possible complications such as infections, pericardial effusion, threshold elevation, lead dislodgment, and lead deficiencies were routinely tracked.

### 2.4 Statistical analysis

Categorical variables in this study were reported as numbers or percentages and were analyzed by Chi‐square test and/or Fischer's exact test whenever appropriate. Continuous variables were reported as mean ± SD and were analyzed by Student's *t* test. All statistical analyses were two‐tailed and a value of *P* < .05 was considered statistically significant. All statistical analyses were performed using SPSS Statistics version 22.0 (Chicago, Illinois).

## 3 RESULTS

### 3.1 Baseline characteristics

Twenty patients with PVs were recruited in the present study. The baseline characteristics are shown in Table 1. The age was 59.2 ± 14.3 years with 11 males in total (11/20, 55%). Three patients had AF with slow heart rate while the rest had a sinus rhythm and AV conduction disease. Right bundle branch block (RBBB) was recorded in three patients, and left bundle branch block (LBBB) was recorded in two patients. Of the cohort, three patients had SAVR, six patients had MVR, four patients had TAVR, one patient had AVR plus MVR, one patient had MVR plus TV ring, two patients had AVR plus MVR and TV ring, and one patient had TVR (supplementary data, Figure S1). Then, 15 patients received dual‐chamber pacemaker implantation and 3 patients received single‐chamber pacemaker implantation.

**TABLE 1:** Baseline characteristics and outcomes

Case	Age	Gender	Valve type	AF	CMP	LVEF	Baseline QRSD (ms)	BBB	Threshold (uni,V@0.4 ms) bundlecapture/Myocardium capture		Sensitivity (uni, mV)	Impendence (uni, Ω)	Paced QRSD (ms)	Stim‐LVAT (ms)
1	54	Male	AVR + MVR		Ischemic	71	135	CRBBB	0.6	0.6	8.2	930	117	62
2	53	Female	AVR			61	95		1.0	0.5	21	875	114	60
3	76	Male	MVR			61	99		2.0	0.4	11.2	657	100	67
4	67	Male	MVR			65	80		0.4	0.4	5.7	455	105	70
5	76	Male	TAVR			74	139	CLBBB	0.6	0.6	14.3	643	107	67
6	53	Female	AVR		Ischemic	36	Paced		0.8	0.8	3.4	385	102	67
7^a^	33	Female	AVR			64	86		—	0.5	20.5	717	149	—
8	53	Male	AVR			61	120		0.6	0.6	7.8	582	107	59
9	50	Female	MVR	AF		73	102		0.4	0.4	8.4	417	92	59
10	68	Female	MVR + TV ring			63	88		0.5	0.5	15	732	102	62
11	61	Male	MVR	AF	Ischemic	59	92		0.6	0.6	14.5	666	104	70
12	46	Female	MVR			62	90		1.4	0.4	15.4	501	106	62
13	74	Female	TAVR			60	144	CRBBB	2.0	0.4	8.2	514	109	69
14	26	Male	TVR			67	109		0.5	0.4	10.9	674	115	76
15	50	Male	MVR + AVR + TV ring	AF		66	88		1.0	0.5	10	820	102	70
16^a^	59	Male	AVR			59	168	CRBBB	—	0.6	9.1	859	142	—
17	50	Female	MVR + AVR + TV ring			67	86		1.2	0.8	12.8	663	118	57
18	60	Male	TAVR		Nonischemic	34	104		0.9	0.3	4.2	518	104	62
19	89	Female	TAVR		Ischemic	47	142	CLBBB	0.5	0.5	13.2	734	106	74
20	57	Male	MVR			54	Paced		0.7	0.7	8	700	107	66

Abbreviations: AF, atrial fibrillation; AVR, aortic valve replacement; CLBBB, complete left bundle branch block; CMP, cardiomyopathy; CRBBB, complete right bundle branch block; LVEF, left ventricular ejection fraction; MVR, mitral valve replacement; PQRSD, paced QRS duration; RVSP, right ventricular septal pacing; stim‐LVAT, stimulus to left ventricular activation time; TAVR, transcatheter aortic valve replacement; TV ring, tricuspid valve ring; TVR, tricuspid valve replacement.

^a^ LBBP failed in cases 7 and 16 and RVSP was performed as an alternative.

### 3.2 Feasibility of LBBP in patients with PVs

**FIGURE 1:**
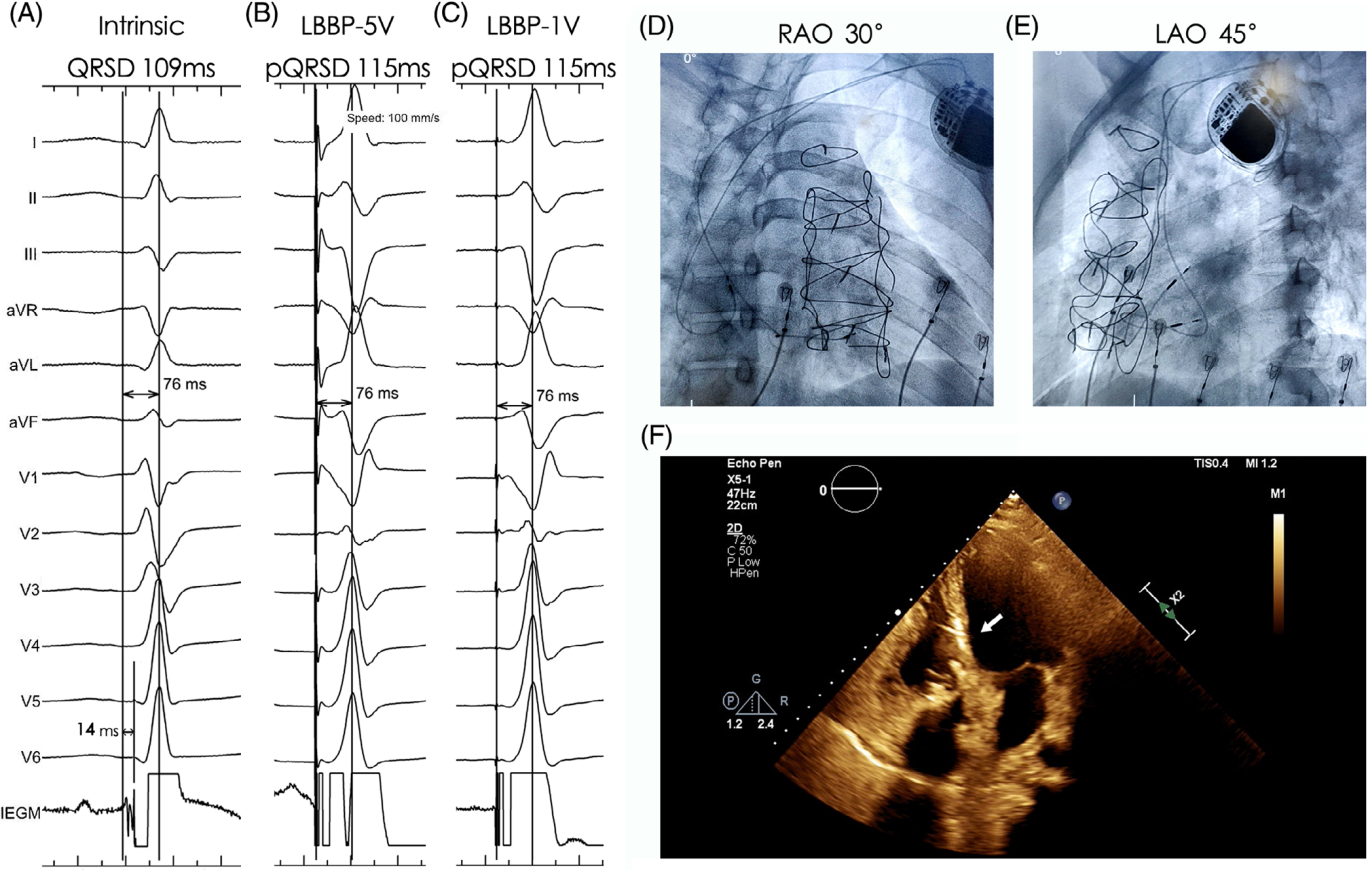
Successful LBBP in a patient after TVR (case 14). The PVI was 14 ms with the intrinsic LVAT being 76 ms, A. The stim‐LVAT was consistent under high output (B, 5 V@0.4 ms) and low output (C, 1 V@0.4 ms). LBBP lead was shown in (D) and (E) under fluoroscopy and in apical four‐chamber view under echo, F. Tricuspid prosthetic valve was visible under echo. LBBP, left bundle branch pacing; PVI, potential to ventricular interval; stim‐LVAT, stimulus to left ventricular activation time; TVR, tricuspid valve replacement

**FIGURE 2:**
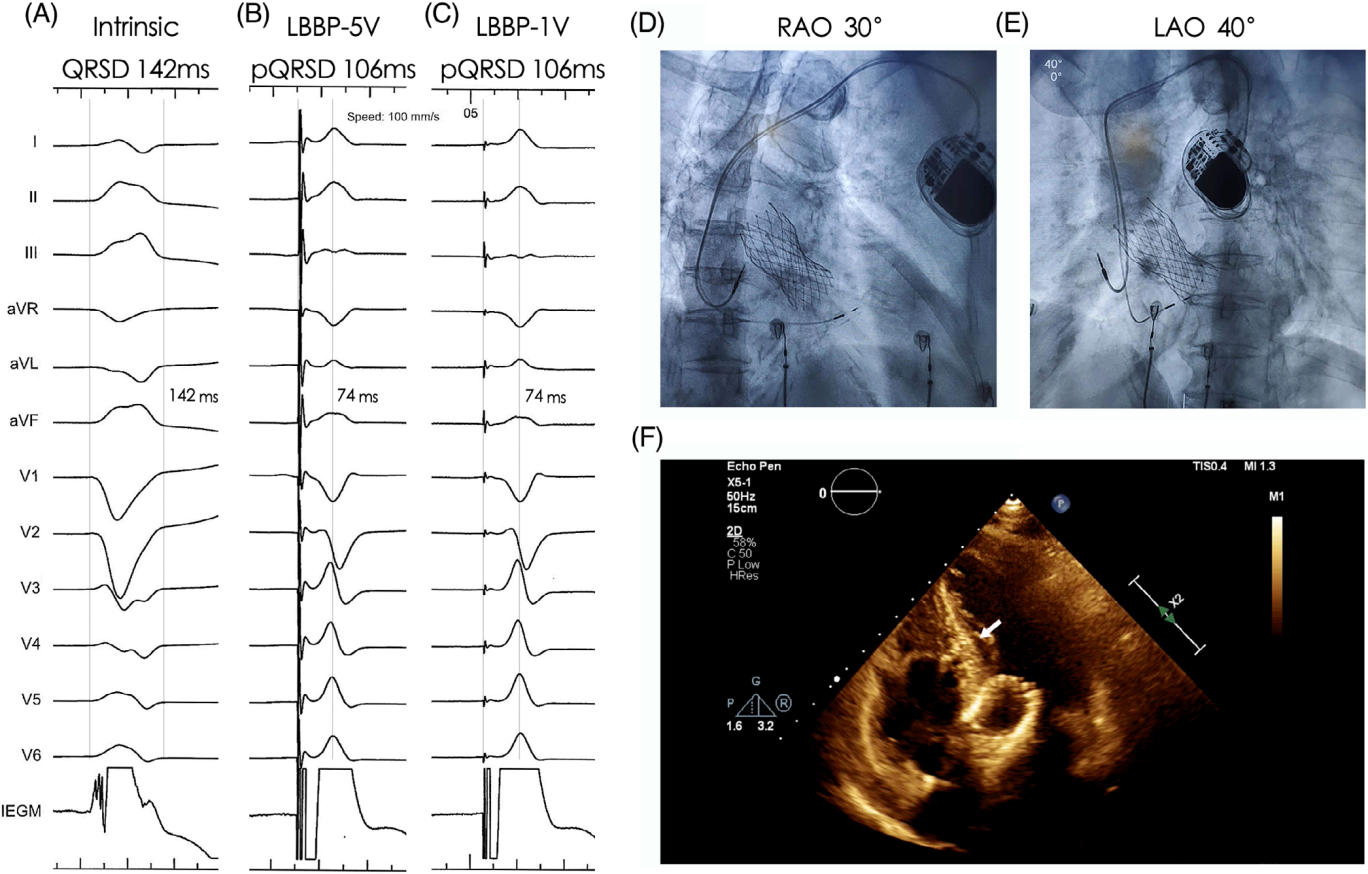
Successful LBBP in a patient with TAVR (case 19). CLBBB morphology was recorded under the intrinsic conduction and no LBB potential was recorded on IEGM, A. The stim‐LVAT was consistent under high output (B, 5 V@0.4 ms) and low output (C, 1 V@0.4 ms), LBBP lead was showed in (D) and (E) under fluoroscopy and in apical five‐chamber view under echo, F. CLBBB, complete left bundle branch block; IEGM, intracardiac electrogram; LBBP, left bundle branch pacing; QRSD, QRS duration; stim‐LVAT, stimulus to left ventricular activation time; TAVR, transcatheter aortic valve replacement

**FIGURE 3:**
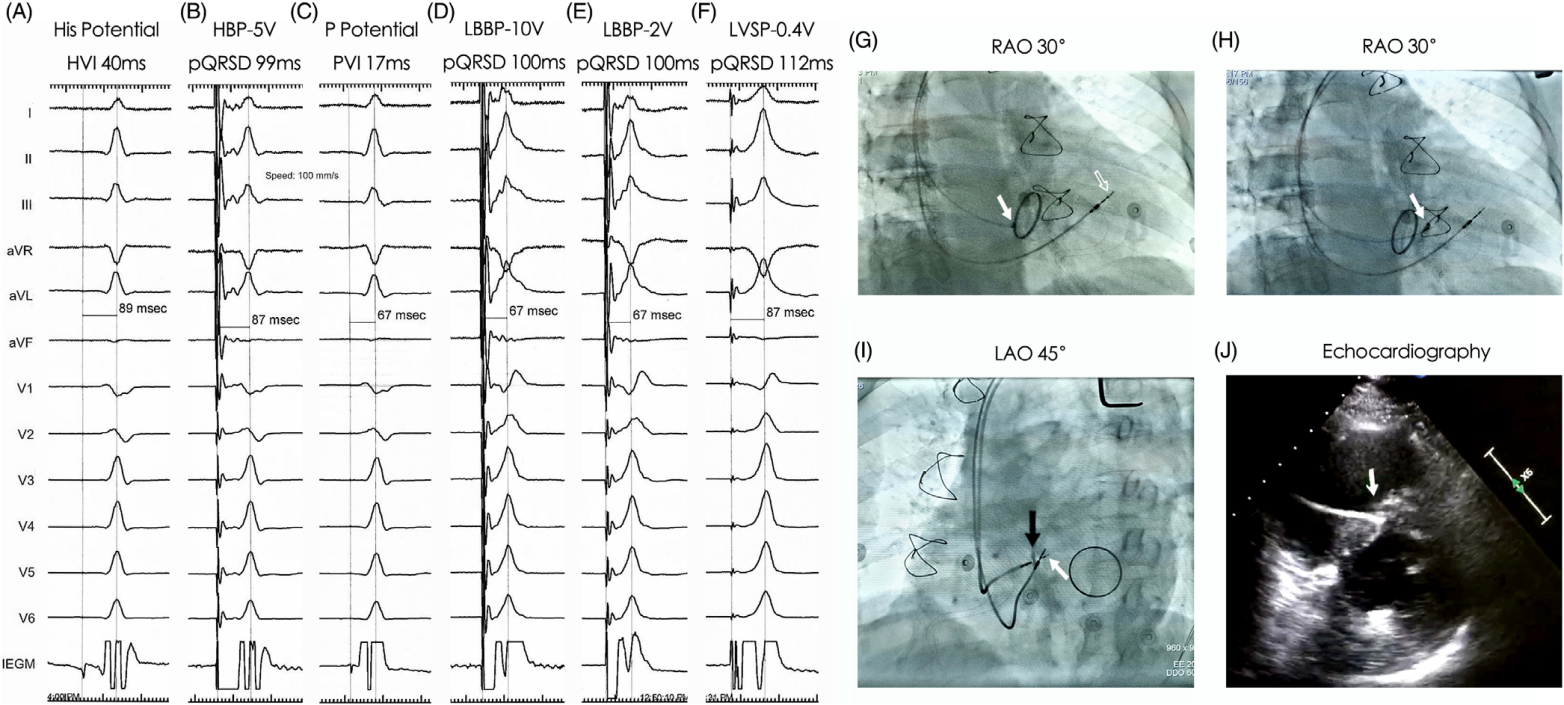
LBBP in a patient with MVR (case 3). His potential was recorded on IEGM with HV of 40 ms and His‐LVAT of 89 ms, A. HBP procedure was attempted, and the stim‐LVAT was 87 ms, B. LBBP procedure was subsequently performed with an LBB potential recorded and the PVI of 17 ms, C. The stim‐LVAT was 67 ms under high output 10 V@0.4 ms, D, and low output 2 V@0.4 ms, E. With the output decreased, LVSP emerged with a stim‐LVAT of 87 ms under the threshold output (0.4 V@0.4 ms, F). His region (solid arrow) and a back‐up RV pacing lead (open arrow) were marked, G, LBBP lead was showed in (H) and (I) under fluoroscopy and in (J) under echo (solid arrow). The depth of LBBP lead in the septum was showed by angiography in (I). HBP, His bundle pacing; HV, his potential to ventricular interval; IEGM, intracardiac electrogram; LBB, left bundle branch; LBBP, left bundle branch pacing; LVSP, left ventricular septal pacing; MVR, mitral valve replacement; pQRSD, paced QRS duration; PVI, potential to ventricular interval; QRSD, QRS duration; RV, right ventricular; stim‐LVAT, stimulus to left ventricular activation time

In the 20 patients with PVs, two patients failed in the LBBP procedure and had traditional RVP instead. Both of them had local hypertrophy secondary to hypertension or aortic stenosis. The mean unipolar bundle capture threshold was 0.84 ± 0.51 V@0.4 ms in the 18 successful participants. The mean fluoroscopic exposure time was 13.0 ± 9.2mins, and radiation dose was 81.7 ± 8.3 mGy. Fluoroscopic exposure time was >30 min in two patients. Between these two patients, one had TVR, and difficulties occurred when advancing the sheath across the tricuspid valvular ring for this patient (Figure 1). For the other patient, significantly enlarged right atrium and cardiac clockwise rotation caused difficulties in placing the lead at a proper position. However, the dual‐lead method was then applied in both patients, resulting in a successful LBBP. New onset of CLBBB was recorded in two patients after TAVR, one of which was intermittent. Successful correction of LBBB and narrowing of QRS duration were documented in these two patients (Figure 2). LBBP was successful in all the patients received MVR (Figure 3).

### 3.3 Surface and IEGM analysis

The QRS duration was 102.6 ± 19.5 ms while the pQRSD was 106.8 ± 6.8 ms. The stim‐LVAT was 65.5 ± 5.4 ms in this cohort. Left bundle branch (LBB) potential was recorded in 61.1% (11/18) patients who succeeded in LBBP, and the amplitude of LBB potential was 0.3 ± 0.1 mV in average. LBB potential could not be recorded in seven patients due to pacing dependency in five (temporary pacing lead was implanted in two before the procedure and three during the procedure) and escape beat morphology of CLBBB in two. The PVI was 21.2 ± 5.8 ms in this cohort. Bundle branch block (BBB) documented in four patients, including two with LBBB, and two with RBBB, was corrected after LBBP.

### 3.4 Procedural and lead‐related complications

Temporary RBBB occurred in two patients with PVs during the procedure and recovered immediately after the procedure. No septal perforation, lead revision, aortic or coronary artery injury, pericardial effusion, or cerebral ischemia was documented in any of the participants. No significant exacerbation of tricuspid or mitral valve regurgitation was recorded either.

### 3.5 Evaluation of lead parameters stability and echocardiographic data

The unipolar myocardial threshold (ventricular capture threshold) was 0.51 ± 0.15v@0.4 ms while the unipolar bundle capture threshold was 0.84 ± 0.51 V@0.4 ms at implantation. Significant decrease in unipolar impedance (428.69 ± 50.61 Ω vs 619.81 ± 153.6 Ω, *P* < .001) and in bipolar impendence (595.88 ± 64.87 Ω vs 803.63 ± 170.91 Ω *P* < 0.001) were documented at 1 month follow‐up in this cohort compared to that during the procedure. The average follow‐up period was 10.4 ± 5.9 months (ranges from 3 to 23 months) in this cohort. Lead parameters, including capture threshold, R wave amplitude and electrode impedance were stable during follow‐up (Table 2). Of the cohort, 15 out of 18 patients fulfilled the 6 months follow‐up and echocardiographic data were documented. No difference in LV end‐diastolic diameter (51.6 ± 7.0 mm vs 48.4 ± 8.3 mm, *P* = .06) and left ventricular ejection fraction (59.1% ± 12.5% vs 58.9% ± 7.4%, *P* = .96) were documented in this cohort compared to that at baseline.

**TABLE 2:** Lead parameters during follow‐up

Time point	Cases	Threshold (V@0.4 ms)		R wave (mv)		Impedance (Ω)	
		Unipolar	Bipolar	Unipolar	Bipolar	Unipolar	Bipolar
Implant	18	0.51 ± 0.15	0.74 ± 0.22	10.73 ± 4.82	12.79 ± 4.78	619.81 ± 153.60*	803.63 ± 170.91*
1 mo	18	0.49 ± 0.12	0.66 ± 0.19	11.81 ± 3.3	14.82 ± 4.32	428.69 ± 50.61	595.88 ± 64.87
3 mo	18	0.52 ± 0.10	0.73 ± 0.18	12.32 ± 3.31	14.80 ± 4.33	421.19 ± 50.94	554.31 ± 112.23
6 mo	15	0.56 ± 0.12	0.83 ± 0.19	12.04 ± 3.82	14.54 ± 4.83	405.56 ± 44.65	563.06. ±76.06
12 mo	6	0.62 ± 0.11	0.79 ± 0.25	12.66 ± 4.51	14.14 ± 3.43	410.67 ± 51.34	562.33 ± 59.12

* *P* < .0001: compared between implant vs each follow‐up visit (1, 3, and 6 months).

## 4 DISCUSSION

In the present study, we observed the feasibility and safety of LBBP in patients with PVs. The main findings are as follows: (a) LBBP was safe and feasible in patients with PVs and (b) lead parameters were stable in patients with PVs after LBBP procedure.

The incidence of postprocedural PPM implantation may range from 4.3% to 30% in different patient populations underwent transcatheter or surgical PV replacement.^3^, ^12^, ^13^, ^14^ This rate can even mount to as high as 39% in patients underwent TAVR with the CoreValve system according to Fraccaro's research.^15^ RVP is the traditional treatment method for patients complicated with CAs after PVs implantation. However, long‐term RVP, especially in patients who have a pacing dependency >40%, may induce electrical and mechanical asynchrony and therefore increase the mortality rate and heart failure hospitalization rate^5^, ^16^ Thus, physiological pacing appears as a more favorable option for this specific patient population.^17^, ^18^

His bundle pacing (HBP) has been a recommended pacing modality for the above‐mentioned patients who have CAs after PVs implantation.^19^, ^20^ Given that few nonspecific intraventricular conduction disease or peripheral conduction block may occur in this specific patient population, HBP may have a unique role, as it realizes electrical synchrony via His‐Purkinje system recruitment and QRS correction. However, the HBP procedure may be a little difficult, especially in patients underwent TAVR. In addition to that, there is a high probability that HBP may lead to rising thresholds and lead revision.^21^ Jonathan et al. reported that LBBB was corrected by HBP in a patient after TAVR, while the output was as high as 5 V@1 ms to correct LBBB at the follow‐up 1 month after the procedure.^19^ Sharma et al confirmed that permanent HBP was feasible in 93% of patients with PVs. However, the success rate for His bundle recruitment was as low as 50% among patients underwent TAVR.^18^ It has been proved that TAVR valve type and depth of implantation are the most important procedural associations with conduction damage.^22^ Pooter et al reported a success rate of 69% in LBBB correction with acceptable pacing thresholds under HBP.^20^ Their results suggested that a more significant involvement of CAs at the level of a more distal site in the conduction system may lead to a lower success rate in HBP.^18^ No wonder that predominantly developed infranodal block are more common in patients with TAVR and lower success rate may be expected during HBP procedure in this cohort. LBBP and HBP have a similar physiological pacing mechanism, which realizes physiological pacing by the rapid recruitment of left His‐Purkinje system. However, a more distal lead fixation in LBB may offer significant advantage in success rate compared with HBP. New onset BBB developed frequently after TAVR or SAVR and associated with increased early and late all‐cause mortality.^6^ It has been demonstrated that patients with typical‐BBB morphology could benefit from LBBP in terms of QRS correction.^23^, ^24^ In the present study, BBB correction via LBBP succeeded in all the four patients complicated with BBB after TAVR or SAVR with a low and stable capture threshold during follow‐up. Therefore, though a relatively small number of patients with BBB were recruited in this study, we still believed that LBBP is more favorable, especially in those patients with aortic valve replacement.

LBBP has been convinced to be feasible and safe in different populations.^25^ Zhang et al confirmed the effectiveness of LBBP in heart failure patients complicated with LBBB.^26^ In our previous study, we convinced that LBBP may bring about better electrical and mechanical synchrony compared with RVP.^7^ Some studies also confirmed the short‐term and medium‐term feasibility and stability of LBBP.^27^, ^28^, ^29^ It has been proved that reduction in electric dyssynchrony was reflected by the shorter QRS duration and stim‐LVAT during LBBP, which could translate to acute hemodynamic effects compared with RVP.^7^, ^27^ In our present study, majority of patients with PVs succeeded in LBBP and presented a narrow pQRSD and short stim‐LVAT. Only two patients failed in LBBP due to hypertrophy secondary to hypertension or aortic stenosis. For these two patients, screwing the lead toward the endocardium in the left ventricular side was still difficult even after multiple attempts, and thus right ventricular septal pacing was performed for them instead. With the instrument improvement, a higher LBBP success rate could be expected in patients with PVs. However, the present study is of a relatively small cohort and of a short observation period, further studies with a larger sample size and a longer observation period are needed to verify the advantages of LBBP and the role of LBBP on mechanical synchrony in the long run in patients with PVs.

PPM procedure may be challenging in patients with TVR. The valve struts may obstruct the access to His bundle region which make a successful HBP impossible in some cases.^30^ Some operators would prefer left ventricular pacing via coronary sinus which may still bring about concerns such as mechanical asynchrony, unsatisfactory pacing thresholds, and subsequent local complications.^31^, ^32^ Insertion of an endocardial lead across a bio‐PV has been proved to be feasible, although it can slightly exacerbate the tricuspid regurgitation, which had been convinced in patients underwent traditional RVP procedure.^32^ LBBP is feasible in patients with TVR. However, the struts of PVs may still limit the ability to steer the lead, and the significantly enlarged right atrium accompanied by cardiac rotation was another challenge that needs to be overcome during LBBP procedure. One patient with tricuspid bio‐PV and three patients with TV ring were recruited in our study. All of them succeeded in the LBBP procedure and no significant aggravation of TV regurgitation was documented in these patients during follow‐up visits. Some operators suggested that intracardiac echo or tricuspid valve annual angiography is helpful in the LBBP procedure.^30^, ^33^ In our opinion, application of adjustable sheath or prefabricating the sheath during procedure was the most important thing in ensuring a successful procedure. With the improvement of instrument, a higher success rate could be expected in the near future in those patients with TVR.

In previous studies, HBP in patients with PVs would also provide a physiologic form of ventricular activation.^18^, ^19^ However, the implementation of HBP has been limited by concerned over suboptimal pacing parameters in this cohort. As described in a previous study, during its mean follow‐up period of 1 year, an increase in capture thresholds has been noted in about 7.4% of patients recruited for resynchronization therapy by HBP.^34^ In the present study, 18 patients underwent LBBP were recruited in this research and the capture threshold was low and stable with optimal pacing parameters throughout the whole follow‐up period. The average follow‐up period was more than 10 months. In light of the lead parameter stability, LBBP is worth prompting in this patient population.

## 5 LIMITATIONS

The present study was a retrospective observational study conducted in a single center and was of a rather short observation period. Randomized prospective studies with a larger sample size and a longer observation period are needed to evaluate the long‐term benefits of LBBP in those patients had PV implantation. In addition, mechanical synchrony should be assessed in future studies to investigate whether the improvement of electrical synchrony can translate to hemodynamic benefits in this patient population, especially in the long run.

## 6 CONCLUSIONS

LBBP was safe and feasible in patients with PVs. Pacing parameters remained stable during the follow‐up period. In terms of success rate and physiological pacing, LBBP was more favorable in patients with TAVR or SAVR.

## CONFLICT OF INTEREST

The authors declare no potential conflict of interests.

## AUTHOR CONTRIBUTIONS

Binni Cai conceived and designed the experiment. Jincun Guo and Linlin Li recruited the subjects and collected the clinical data. Xinyi Huang, Qiang Li, and Linlin Li conducted the laboratory testing. Guosheng Xiao and Yan Wang helped analyze the data. Jincun Guo and Linlin Li wrote the manuscript. All authors read and approved the final manuscript.

## ETHICS STATEMENT

The study was approved by the Ethics Committee of Xiamen Cardiovascular Hospital, Xiamen University and was performed in line with the principles of the Declaration of Helsinki. The institutional review board waived the need of written patient informed consent.

## Supporting information


**Figure S1** Patients with PVs recruited in the study. 18 patients succeeded in LBBP and 2 patients failed, both of whom had AVR. AVR: aortic valve replacement; MVR: mitral valve replacement; TAVR: transcatheter aortic valve replacement; TVR: tricuspid valve replacement; TV ring: tricuspid valve ring; PV: prosthetic valve; LBBP: left bundle branch pacing.

## Data Availability

The datasets generated and/or analyzed during the current study are available from the corresponding author, Binni Cai, upon reasonable request.
