# Supplementary figures and images for: Feasibility and stability of left bundle branch pacing in patients after prosthetic valve implantation
Source: Clin Cardiol. 2020 Jul 1;43(10):1110–8. doi: 10.1002/clc.23413 (PMC7533988; doi:10.1002/clc.23413)

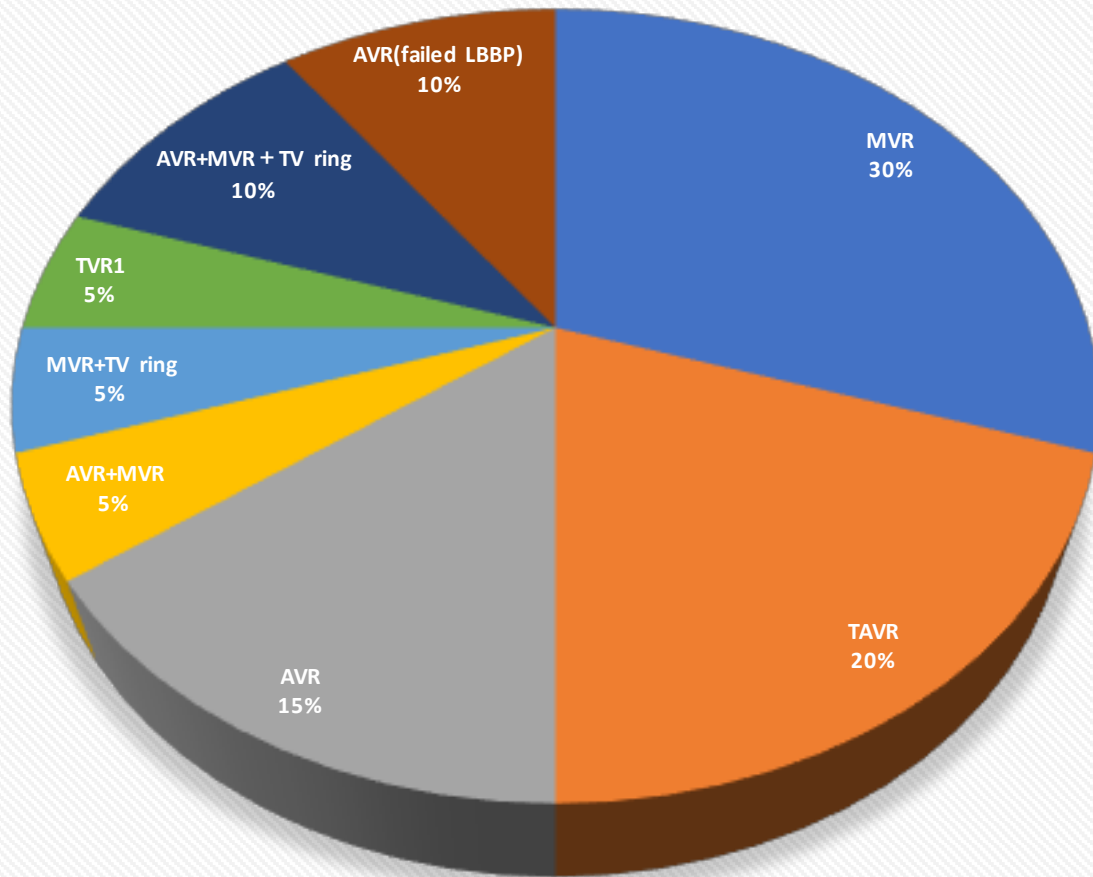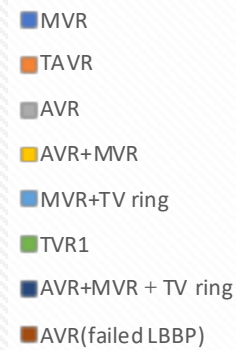

Supplement: Supplementary file 1 — Figure S1 Patients with PVs recruited in the study. 18 patients succeeded in LBBP and 2 patients failed, both of whom had AVR. AVR: aortic valve replacement; MVR: mitral valve replacement; TAVR: transcatheter aortic valve replacement; TVR: tricuspid valve replacement; TV ring: tricuspid valve ring; PV: prosthetic valve; LBBP: left bundle branch pacing. [file CLC-43-1110-s001.pdf]
